# Cross-sectional analysis of periodontitis and peripheral artery disease association: Results from National Health and Nutrition Examination Survey 1999 to 2004

**DOI:** 10.1097/MD.0000000000048117

**Published:** 2026-03-20

**Authors:** Xin Li, Suhong Wu, Meisheng Zou

**Affiliations:** a Department of Endodontics and Operative Dentistry, Hospital of Stomatology, Zhongshan, Guangdong, China; b Department of Geriatrics, Zhongshan City People’s Hospital, Zhongshan, Guangdong, China.

**Keywords:** cross-sectional study, NHANES, PAD, periodontitis

## Abstract

Utilizing 1999 to 2004 National Health and Nutrition Examination Survey (NHANES) datasets, this cross-sectional analysis evaluates the relationship of periodontitis with peripheral arterial disease (PAD) among adults in the United States. Our investigation employed NHANES 1999 to 2004 datasets to quantify the periodontitis-PAD association. Clinical classifications applied Center for Disease Control and the American Academy of Periodontics (CDC/AAP) diagnostic thresholds. An ankle-brachial index below 0.9 in any leg defined PAD. Multivariable logistic regression quantified this relationship, complemented with stratified subgroup assessments for robustness verification. Within the cohort of 4133 individuals (mean ± standard deviation age: 56.8 ± 12.1 years; female proportion: 46.8%), periodontal disease affected 16.7% (n = 690). PAD prevalence reached 4.4% (n = 182). Comparative analysis revealed elevated PAD frequency in periodontitis subjects versus non-periodontitis controls (8.1% [56/690] vs 3.7% [126/3443]). Following comprehensive covariate adjustment, periodontitis maintained independent association with PAD (OR = 1.49; 95% CI:1.04–2.13; *P*-value = .03). Stratified analyses indicated absence of significant interaction effects (all interaction *P*-values > .05). Analysis of this nationally representative American population reveals periodontitis as an independent predictor of heightened PAD probability. Prospective investigations remain imperative to determine causality.

## 1. Introduction

Periodontal disease is characterized by progressive degradation of tooth-supporting structures.^[1]^ Surveillance data demonstrate that approximately half of American adults ≥ 30 years manifest periodontal disease, including severe forms in 10% of this demographic.^[2]^ Worldwide, advanced periodontitis afflicts nearly 11% of the global population.^[3]^ Contemporary studies link this oral condition to multiple systemic pathologies,^[4]^ encompassing osteoporosis, chronic renal impairment, diabetes mellitus, dyslipidaemia, and cardiovascular disorders (CVD).^[5–9]^ The World Health Organization considers periodontal health integral to overall wellbeing.^[10]^

Characterised by atherosclerotic links, peripheral arterial disease (PAD) presents significant luminal narrowing in peripheral arteries, where intermittent claudication constitutes the primary clinical manifestation.^[11]^ Shared pathophysiological pathways between PAD and CVD involve systemic hyperinflammation as a key pathogenic driver.^[12]^ Elevated circulating inflammatory mediators are documented to correlate with advancement of both CVD and PAD.^[13–15]^ Although robust evidence confirms periodontitis-CVD connections,^[16–20]^ mechanistic similarities exist among these disorders: all represent chronic inflammatory conditions with overlapping mediator profiles. This pathobiological convergence has stimulated focused investigation into periodontitis-PAD linkages. Mendez et al^[21]^ established that baseline periodontitis conferred 2.27-fold increased probability of incident PAD (OR = 2.27; 95% CI = 1.32–3.90). Subsequent case-control research by Soto-Barreras et al^[22]^ documented a notable periodontitis-PAD association (OR = 8.18; 95% CI = 1.21–35.23). Consistent evidence continues to accumulate.^[23–25]^ Notwithstanding these findings, methodological constraints require Acknowledgments: multiple investigations possess insufficient statistical power, while others predominantly examine European/Asian demographics with inadequate representation of American cohorts. Thus, comprehensive population studies in US settings remain essential to validate this relationship.

To mitigate these constraints, we executed a thorough examination of the periodontitis-PAD linkage among US adults employing National Health and Nutrition Examination Survey (NHANES) 1999 to 2004 datasets. This survey offers a methodologically robust framework for epidemiological inquiry via nationally representative sampling and standardized clinical evaluations. Our investigation statistically determined this relationship following confounder adjustment, with analytical robustness verified through stratified subgroup assessments.

## 2. Materials and methods

### 2.1. Data and sample source

**Figure 1.**
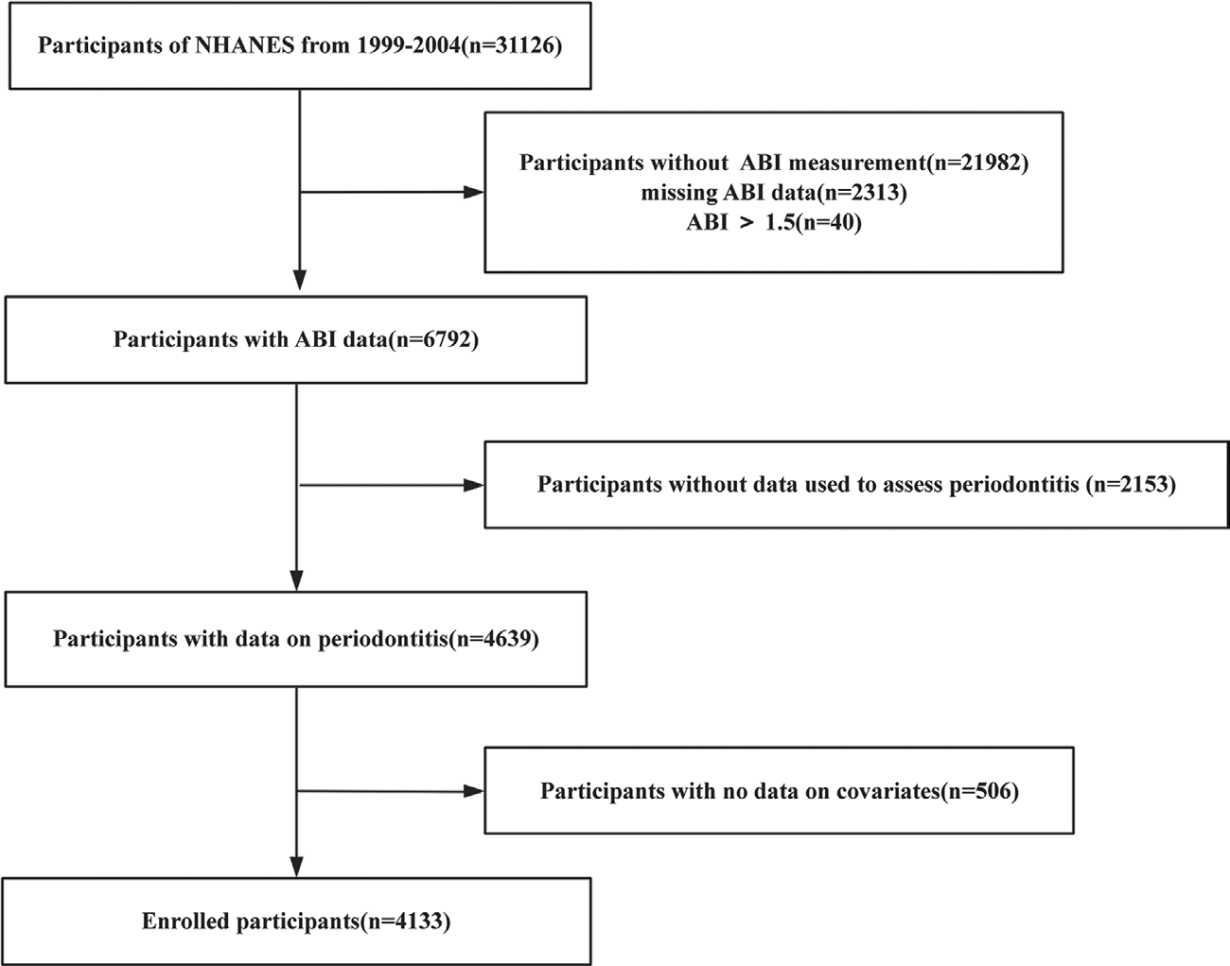
Flowchart showing the selection process of study population.

The study analyzed data obtained from the 1999 to 2004 cycles of the NHANES. NHANES is a nationally representative surveillance system that monitors health and nutritional indicators among the non-institutionalized U.S. population,^[26]^ utilizing a multi-stage stratified probability sampling approach.^[27]^ Publicly available NHANES datasets, provided by the National Center for Health Statistics with approval from institutional review boards, include extensive demographic, socioeconomic, dietary, and health-related information. Prior to enrollment, all subjects provided written informed consent. Our research team performed a secondary analysis using de-identified NHANES information (accessible at: http://www.cdc.gov/nchs/nhanes/), which does not necessitate further ethical clearance. From an initial pool of 31,126 individuals, exclusions were made for those lacking ankle-brachial index (ABI) readings, incomplete periodontal disease data, missing covariate values, or ABI exceeding 1.5 (suggestive of arterial stiffness, n = 40), resulting in a final analytical sample of 4133 participants (Fig. 1).

### 2.2. Assessment of periodontitis

Periodontal assessments in the NHANES 1999 to 2004 “Oral Health-Periodontal Screening” component involved recording measurements at 6 sites per tooth (up to 28 teeth). Two clinical indicators, clinical attachment loss (CAL) and probing depth (PD), were captured during this evaluation. Case classification for periodontitis aligned with the Center for Disease Control and the American Academy of Periodontics (CDC/AAP) criteria.^[28,29]^ Severe disease was characterized by the presence of ≥2 interproximal sites exhibiting CAL ≥ 6 mm (on non-adjacent teeth) accompanied by ≥1 interproximal site with PD ≥ 5 mm. Moderate periodontitis was categorized as meeting either of the following conditions: ≥2 interproximal sites with PD ≥ 5 mm located on distinct teeth, or ≥2 interproximal sites demonstrating CAL ≥ 4 mm on separate teeth.^[28,29]^ Individuals diagnosed with moderate or severe periodontitis were designated as the case group; conversely, participants exhibiting no or mild disease comprised the comparator group.^[30,31]^

### 2.3. Measurement of ABI and PAD

PAD, the primary endpoint, was operationalized as an ABI value below 0.9.^[32,33]^ Standardized procedures for ABI assessment in NHANES were rigorously followed.^[34]^ In brief, systolic blood pressure readings were obtained from the right brachial artery and bilateral posterior tibial arteries using appropriate cuffs in eligible participants meeting weight criteria (≤181 kg). Participants aged 40 to 59 years had 2 measurements taken per site, which were then averaged; those 60 years or older received a single reading per site. If contraindications existed for right arm measurement, left brachial pressure served as the substitute. The ABI value was derived by dividing the mean ankle systolic pressure by the brachial systolic pressure, utilizing the lower of the 2 ankle pressure averages for each leg in subsequent analyses. Individuals displaying ABI ≥ 1.5 were excluded.

### 2.4. Covariates

Potential confounders in this analysis were selected based on: factors documented in prior research to influence both periodontitis and PAD^[9,35–37]^; conformity with STROBE statement recommendations^[38]^; and covariates inducing a ≥10% alteration in the initial regression coefficient for the association between periodontitis and PAD, which were deemed significant confounders. Accordingly, the following covariates, meeting the above criteria, were included: age (years), sex (male, female), education (<high school, high school, and >high school), poverty income ratio (PIR: ≤1.3, 1.3–3.5, ≥3.5), body mass index (BMI, kg/m^2^), smoking (never, former, current), drinking (never, former, current) and PA (low, moderate, high). Then, comorbidities including diabetes (yes or no), hypertension (yes or no), hyperlipidemia (yes or no), CVD such as coronary heart disease (yes or no), congestive heart failure (yes or no), angina (yes or no) and stroke (yes or no).

### 2.5. Statistical analysis

Categorical variables are presented as proportions, continuous variables as mean ± standard deviation. Intergroup differences were assessed using χ^2^ tests for categorical data and Student *t* tests for continuous variables. Multivariate logistic regression was used to analyze the association between periodontitis and PAD in 3 different models. In Model 1, no covariates were adjusted. Model 2 adjusted for age and sex. Model 3 adjusted for age, sex, education, PIR, BMI, smoking status, drinking status, PA, hypertension, hyperlipidemia, diabetes and CVD. Subgroup analysis of the association between periodontitis and PAD was conducted with stratified factors including sex (male/female), age (<65/≧65), BMI (<30/≧30), smoking (never, former, current), drinking (never, former, current), diabetes (yes/no), hypertension (yes/no), hyperlipidemia (yes/no) and CVD (yes or no). Additionally, an interaction term was conducted to assess subgroup heterogeneity. All analyses were performed using R software, and statistical significance was set at *P* < .05 (two-sided).

## 3. Results

### 3.1. Participant characteristics

Table 1 details the demographic and clinical profiles of the 4133 study subjects (mean age: 56.8 ± 12.1 years; male: 53.2%, female: 46.8%). Individuals were categorized based on periodontal health status into a non-periodontitis cohort (n = 3443) and a periodontitis cohort (n = 690). Overall PAD prevalence reached 4.4% (n = 182), with a significantly elevated prevalence among those with periodontitis relative to periodontally healthy subjects (8.1% [56/690] vs 3.7% [126/3443]). Significant disparities emerged across groups (*P* < .05) for all evaluated variables except PA level (PA; *P* = .282) and hyperlipidemia (*P* = .626), encompassing age, sex, poverty income ratio (PIR), educational attainment, body mass index (BMI), smoking status, alcohol intake, cardiovascular disease (CVD), hypertension, and diabetes mellitus.

**Table 1:** Baseline characteristics of the study participants.

Variables	Total (n = 4133)	No periodontitis (n = 3443)	Periodontitis (n = 690)	*P*
Age (yr)	56.8 ± 12.1	55.9 ± 11.8	61.0 ± 12.5	<.001
Sex, n (%)				
Male	2198 (53.2)	1734 (50.4)	464 (67.2)	<.001
Female	1935 (46.8)	1709 (49.6)	226 (32.8)	
PIR, n (%)				
≤1.3	910 (22.0)	680 (19.8)	230 (33.3)	<.001
1.3–3.5	1504 (36.4)	1214 (35.3)	290 (42)	
≥3.5	1719 (41.6)	1549 (45)	170 (24.6)	
Education, n (%)				
Below high school level	1157 (28.0)	861 (25)	296 (42.9)	<.001
High school level	955 (23.1)	794 (23.1)	161 (23.3)	
Above school level	2021 (48.9)	1788 (51.9)	233 (33.8)	
Smoking, n (%)				
Never	2019 (48.9)	1800 (52.3)	219 (31.7)	<.001
Former	1326 (32.1)	1073 (31.2)	253 (36.7)	
Now	788 (19.1)	570 (16.6)	218 (31.6)	
Drinking, n (%)				
Never	541 (13.1)	462 (13.4)	79 (11.4)	<.001
Former	870 (21.1)	685 (19.9)	185 (26.8)	
Now	2722 (65.9)	2296 (66.7)	426 (61.7)	
PA, n (%)				
Low	2906 (70.3)	2417 (70.2)	489 (70.9)	.282
Moderate	972 (23.5)	821 (23.8)	151 (21.9)	
High	255 (6.2)	205 (6)	50 (7.2)	
BMI (kg·m^2^)	28.4 ± 5.6	28.5 ± 5.5	28.0 ± 5.8	.016
CVD, n (%)				
No	3795 (91.8)	3189 (92.6)	606 (87.8)	<.001
Yes	338 (8.2)	254 (7.4)	84 (12.2)	
Hyperlipidemia, n (%)				
No	941 (22.8)	779 (22.6)	162 (23.5)	.626
Yes	3192 (77.2)	2664 (77.4)	528 (76.5)	
Hypertension, n (%)				
No	2142 (51.8)	1863 (54.1)	279 (40.4)	<.001
Yes	1991 (48.2)	1580 (45.9)	411 (59.6)	
Diabetes, n (%)				
No	3528 (85.4)	2987 (86.8)	541 (78.4)	<.001
Yes	605 (14.6)	456 (13.2)	149 (21.6)	
PAD, n (%)				
No	3951 (95.6)	3317 (96.3)	634 (91.9)	<.001
Yes	182 (4.4)	126 (3.7)	56 (8.1)	

BMI = body mass index, CVD = cardiovascular disease, PA = physical activity, PAD = peripheral arterial disease, PIR = family income to poverty ratio.

### 3.2. Associations between periodontitis and PAD

The association between periodontitis and PAD was quantified employing multivariable logistic regression, implementing sequential adjustments for covariates. Model 1 (unadjusted) revealed that individuals with periodontitis had 2.33 times the odds of PAD compared to periodontally healthy subjects (OR = 2.33; 95% CI: 1.68–3.22; *P* < .001). Following adjustment for age and sex (Model 2), this positive correlation remained statistically significant (OR = 1.75; 95% CI: 1.25–2.47; *P* = .01). After extensive adjustment for demographic, behavioral, and clinical covariates – including smoking status, body mass index (BMI), alcohol intake, physical activity (PA), educational attainment, PIR, hypertension, hyperlipidemia, cardiovascular disease (CVD), and diabetes mellitus (Model 3) – periodontitis maintained an independent link to PAD (OR = 1.49; 95% CI: 1.04–2.13; *P* = .03). Detailed regression outputs are provided in Table 2.

**Table 2:** Association between periodontitis and peripheral arterial disease among participants in National Health and Nutritional Examination Surveys 1999–2004.

Exposure	Model 1 (OR, 95% CI)	*P*–value	Model 2 (OR, 95% CI)	*P*–value	Model 3 (OR, 95% CI)	*P*–value
Periodontitis						
No	1.0	–	1.0	–	1.0	–
Yes	2.33 (1.68–3.22)	<.001	1.75 (1.25–2.47)	.001	1.49 (1.04–2.13)	.03

Model 1 was adjusted for none; Model 2 was adjusted for age, sex; Model 3 was adjusted for age, sex, smoking, BMI, drinking, PA, education, PIR, hypertension, hyperlipidemia, CVD, and diabetes.

BMI = body mass index, CI = confidence intervals, CVD = cardiovascular disease, OR = odds ratio, PA = physical activity, PIR = family income to poverty ratio.

### 3.3. Subgroup analysis and interaction test of periodontitis and PAD

**Figure 2.**
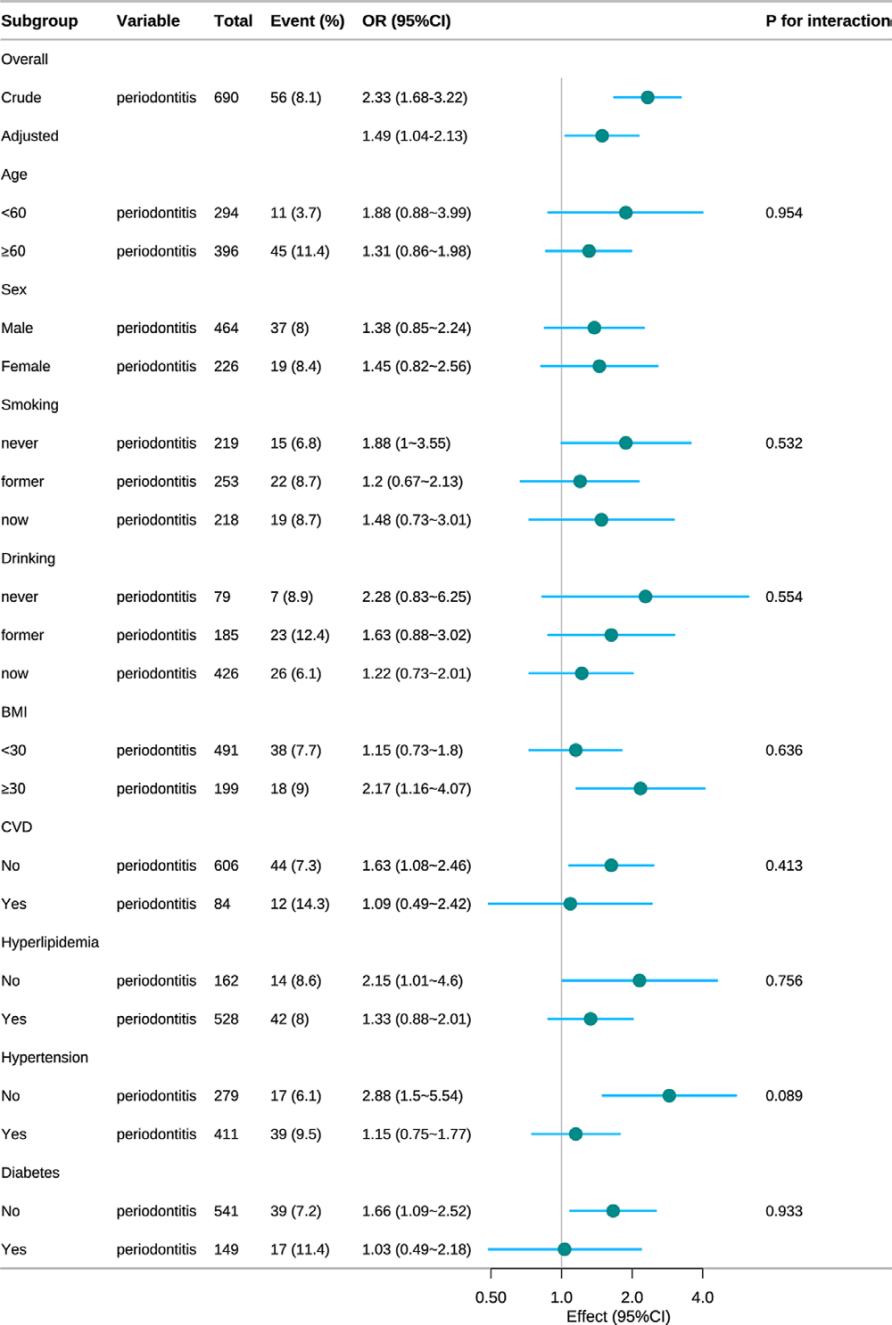
Subgroup analysis of the association between periodontitis and peripheral arterial disease in study population from 1999–2004 NHANES. BMI = body mass index, CI = confidence interval, CVD = cardiovascular disease, OR = odds ratio.

The robustness of our findings was further assessed via stratified subgroup analyses (Fig. 2). Tests for interaction within demographic and clinical subgroups – specifically age, sex, smoking status, alcohol intake, body mass index (BMI), hypertension, hyperlipidemia, cardiovascular disease (CVD), and diabetes mellitus – showed no statistically significant effect modifications (all *P*-interaction > .05). Notably, a consistent positive association between periodontitis and PAD was observed across every subgroup evaluated.

## 4. Discussion

This extensive cross-sectional investigation, utilizing data from the 1999 to 2004 NHANES, reveals a significant positive link connecting periodontitis and PAD in the US adult population, even after comprehensive adjustment for covariates. Analyses across subgroups further supported the persistence of this relationship, with interaction analyses revealing stable effects throughout diverse demographic and clinical subgroups.

Earlier meta-analytic research indicated a positive relationship between periodontitis and PAD within Asian and European cohorts.^[39]^ Ahn and colleagues^[40]^ identified periodontitis as a PAD risk factor, reporting a 2.03-fold elevated risk (95% CI = 1.05–3.93) among affected individuals. A case-control investigation by Chen et al^[41]^ determined that periodontitis conferred a reltive PAD risk of 5.45 (95% CI = 1.57–18.89). Çalapkorur et al^[42]^ demonstrated that periodontitis elevated the odds ratio for PAD development to 5.84 (95% CI = 1.56–21.91). Nevertheless, investigations involving the US population remain limited. Our analysis revealed an increased PAD risk among periodontitis patients (adjusted OR = 1.67; 95% CI: 1.03–2.70) after accounting for age, sex, smoking, BMI, alcohol consumption, PA, educational attainment, PIR, hypertension, hyperlipidemia, CVD, and diabetes mellitus. To date, only 2 US-based studies have explored this association. One investigation,^[21]^ utilizing 1998 data from the Normative Aging Study and Dental Longitudinal Study (US Department of Veterans Affairs), documented an OR of 2.27 (95% CI: 1.32–3.9) for the periodontitis-PAD link. The other study^[22]^ observed a positive correlation (OR = 8.18; 95% CI: 1.21–35.23). These outcomes are congruent with our findings. Significantly, previous studies did not evaluate associations within specific subgroups nor interaction effects across demographic and clinical strata. Our stratified analyses substantiate the consistent stability of the association across all evaluated subgroups.

The observed link between periodontitis and PAD likely encompasses both indirect and direct pathogenic routes. Systemic increases inflammatory mediators constitute a primary indirect pathway: cytokines originating from periodontal disease sites (e.g., interleukins [ILs] and tumor necrosis factor-alpha [TNF-α]) may promote endothelial dysfunction, facilitating atherosclerosis progression and potentially fostering PAD development.^[43,44]^ Direct pathways could include dissemination of periodontal pathogens through transient bacteremia induced by routine oral activities. Following entry into the bloodstream, these microbes – transported inside phagocytes or circulating freely – can be deposited within atheromatous plaques, possibly aggravating vascular damage.^[45–47]^

This study possesses several key strengths: the use of NHANES data confers considerable statistical power via its nationally representative design; extensive control for recognized PAD risk factors; and thorough stratified analyses incorporating covariate adjustment, bolstering methodological rigor. Notwithstanding these merits, certain limitations require Acknowledgments. First, the cross-sectional nature of the study hinders establishing temporal precedence between periodontitis and PAD – prospective cohort investigations are crucial for inferring causality. Second, residual confounding attributable to unassessed variables might remain. Lastly, limited ABI data availability for individuals under 40 years old constrains age-distribution analyses, though this mirrors PAD’s higher prevalence in older adults.

## 5. Conclusion

In summary, this study confirms a robust and independent link connecting periodontitis with the prevalence of PAD. Prospective longitudinal investigations are crucial to verify the causal direction underlying this observed association.

## Acknowledgments

This study used data from the NHANES. The authors would like to thank all contributors and participants in the NHANES.

## Author contributions

**Formal analysis:** Xin Li.

**Funding acquisition:** Xin Li, Suhong Wu.

**Investigation:** Xin Li.

**Methodology:** Xin Li.

**Project administration:** Suhong Wu.

**Resources:** Suhong Wu.

**Software:** Suhong Wu, Meisheng Zou.

**Supervision:** Meisheng Zou.

**Validation:** Meisheng Zou.

**Writing – original draft:** Xin Li.

**Writing – review & editing:** Meisheng Zou.

## References

[1] Xie L Chen Q Xu H Li C Lu J Zhu Y. The research progress on periodontitis by the National Natural Science Foundation of China. Int J Oral Sci. 2025;17:44. 40461458 10.1038/s41368-025-00371-x PMC12134109

[2] Eke PI Zhang X Lu H. Predicting periodontitis at state and local levels in the United States. J Dent Res. 2016;95:515–22. 26848071 10.1177/0022034516629112 PMC6092742

[3] Jiao J Jing W Si Y. The prevalence and severity of periodontal disease in Mainland China: data from the fourth national oral health survey (2015–2016). J Clin Periodontol. 2021;48:168–79. 33103285 10.1111/jcpe.13396

[4] Bashir NZ Woolf BAR Burgess S Bernabé E. Periodontitis and systemic disease: the impact of covariate selection. J Dent Res. 2025;105:59–66. 40676930 10.1177/00220345251356469 PMC12701905

[5] Tilotta F Gosset M Herrou J Briot K Roux C. Association between osteoporosis and periodontitis. Joint Bone Spine. 2025;92:105883. 40090617 10.1016/j.jbspin.2025.105883

[6] Parsegian K Randall D Curtis M Ioannidou E. Association between periodontitis and chronic kidney disease. Periodontol 2000. 2022;89:114–24. 35244955 10.1111/prd.12431

[7] Zhu J Xu W Wu S Song D. Vitamin B6 status, type 2 diabetes mellitus, and periodontitis: evidence from the NHANES database 2009–2010. BMC Oral Health. 2025;25:299. 39994649 10.1186/s12903-025-05597-z PMC11852513

[8] Xu J Zhang R Lin S. Association between periodontitis with the all-cause and cause specific mortality among the population with hyperlipidemia. BMC Oral Health. 2024;24:1246. 39427172 10.1186/s12903-024-05055-2 PMC11490175

[9] Zhang Z Zhao X Gao S. Biological aging mediates the association between periodontitis and cardiovascular disease: results from a national population study and Mendelian randomization analysis. Clin Epigenetics. 2024;16:116. 39182082 10.1186/s13148-024-01732-9 PMC11344936

[10] Petersen PE Kwan S. The 7th WHO global conference on health promotion: towards integration of oral health (Nairobi, Kenya, 2009). Community Dent Health. 2010;27(Suppl 1):1–9.

[11] Kansal A Davis AM Rymer JA. Management of peripheral artery disease. JAMA. 2025;334:444. 40601325 10.1001/jama.2025.8408

[12] Cicero AFG Salvetti M. Peripheral artery disease: a highly prevalent untreated and uncontrolled independent cardiovascular disease. Atherosclerosis. 2022;354:55–6. 35791966 10.1016/j.atherosclerosis.2022.06.1015

[13] Chan NC Xu K de Vries TAC Eikelboom JW Hirsh J. Inflammation as a mechanism and therapeutic target in peripheral artery disease. Can J Cardiol. 2022;38:588–600. 35114347 10.1016/j.cjca.2022.01.026

[14] Levin MG Klarin D Georgakis MK; VA Million Veteran Program. A missense variant in the IL-6 receptor and protection from peripheral artery disease. Circ Res. 2021;129:968–70. 34547901 10.1161/CIRCRESAHA.121.319589 PMC8556352

[15] Arnold N Koenig W. Inflammation in atherosclerotic cardiovascular disease: from diagnosis to treatment. Eur J Clin Invest. 2025;55:e70020. 40055964 10.1111/eci.70020 PMC12169090

[16] Carra MC Rangé H Caligiuri G Bouchard P. Periodontitis and atherosclerotic cardiovascular disease: a critical appraisal. Periodontol 2000. 2023:1–34. 10.1111/prd.12528 37997210

[17] Lai Y Liu S Song C Long T Song L Jiang M. An update on the role and mechanisms of periodontitis in cardiovascular diseases. Cell Signal. 2025;132:111770. 40164419 10.1016/j.cellsig.2025.111770

[18] Molina A Ambrosio N Molina M. Effect of periodontal therapy on endothelial function and serum biomarkers in patients with periodontitis and established cardiovascular disease: a pilot study. Front Oral Health. 2025;6:1488941. 39996093 10.3389/froh.2025.1488941 PMC11847872

[19] Yang X Luo M Jiang Y. The regulatory effect of zinc on the association between periodontitis and atherosclerotic cardiovascular disease: a cross-sectional study based on the National Health and Nutrition Examination Survey. BMC Oral Health. 2024;24:703. 38890599 10.1186/s12903-024-04473-6 PMC11184828

[20] Ye Z Cao Y Miao C. Periodontal therapy for primary or secondary prevention of cardiovascular disease in people with periodontitis. Cochrane Database Syst Rev. 2022;10:CD009197. 36194420 10.1002/14651858.CD009197.pub5 PMC9531722

[21] Mendez MV Scott T LaMorte W Vokonas P Menzoian JO Garcia R. An association between periodontal disease and peripheral vascular disease. Am J Surg. 1998;176:153–7. 9737622 10.1016/s0002-9610(98)00158-5

[22] Soto-Barreras U Olvera-Rubio JO Loyola-Rodriguez JP. Peripheral arterial disease associated with caries and periodontal disease. J Periodontol. 2013;84:486–94. 22680302 10.1902/jop.2012.120051

[23] Bloemenkamp DG van den Bosch MA Mali WP. Novel risk factors for peripheral arterial disease in young women. Am J Med. 2002;113:462–7. 12427494 10.1016/s0002-9343(02)01258-5

[24] Cho DH Song IS Choi J Gwon JG. Risk of peripheral arterial disease in patients with periodontitis: a nationwide, population-based, matched cohort study. Atherosclerosis. 2020;297:96–101. 32109666 10.1016/j.atherosclerosis.2020.02.012

[25] Aoyama N Suzuki JI Kobayashi N. Periodontitis deteriorates peripheral arterial disease in Japanese population via enhanced systemic inflammation. Heart Vessels. 2017;32:1314–9. 28567552 10.1007/s00380-017-1003-6

[26] Fain JA. NHANES. Diabetes Educ. 2017;43:151. 28340543 10.1177/0145721717698651

[27] Johnson CL Dohrmann SM Burt VL Mohadjer LK. National Health and Nutrition Examination Survey: sample design, 2011–2014. Vital Health Stat. 2014;162:1–33. 25569458

[28] Eke PI Page RC Wei L Thornton-Evans G Genco RJ. Update of the case definitions for population-based surveillance of periodontitis. J Periodontol. 2012;83:1449–54. 22420873 10.1902/jop.2012.110664 PMC6005373

[29] Eke PI Dye BA Wei L. Update on prevalence of periodontitis in adults in the United States: NHANES 2009 to 2012. J Periodontol. 2015;86:611–22. 25688694 10.1902/jop.2015.140520 PMC4460825

[30] ALHarthi SSY Natto ZS Midle JB. Association between time since quitting smoking and periodontitis in former smokers in the National Health and Nutrition Examination Surveys (NHANES) 2009 to 2012. J Periodontol. 2019;90:16–25. 30102767 10.1002/JPER.18-0183

[31] Hou K Zhang H Song W Li S Liu J Ma Z. Association between life’s essential 8 and periodontitis: a study based on NHANES 2009–2014. Front Med (Lausanne). 2024;11:1342792. 38681053 10.3389/fmed.2024.1342792 PMC11045882

[32] Menke A Muntner P Wildman RP Dreisbach AW Raggi P. Relation of borderline peripheral arterial disease to cardiovascular disease risk. Am J Cardiol. 2006;98:1226–30. 17056334 10.1016/j.amjcard.2006.05.056

[33] Selvin E Erlinger TP. Prevalence of and risk factors for peripheral arterial disease in the United States: results from the National Health and Nutrition Examination Survey, 1999–2000. Circulation. 2004;110:738–43. 15262830 10.1161/01.CIR.0000137913.26087.F0

[34] Yu Z Lu B Han R Tu C. Exploring the hemoglobin-to-red blood cell distribution width ratio (HRR) to peripheral arterial disease nexus: a comprehensive analysis of NHANES data from 1999 to 2004. Front Pharmacol. 2025;16:1529155. 39911849 10.3389/fphar.2025.1529155 PMC11794118

[35] Li J Yao Y Yin W. Association of periodontitis with cardiovascular and all-cause mortality in hypertensive individuals: insights from a NHANES cohort study. BMC Oral Health. 2024;24:950. 39152381 10.1186/s12903-024-04708-6 PMC11328503

[36] Song Q Zhang H Su Y Song J. The link between periodontitis and atherosclerotic cardiovascular disease in non-Hispanic White adults: NHANES 1999 to 2014. PLoS One. 2025;20:e0321220. 40299909 10.1371/journal.pone.0321220 PMC12040079

[37] Kadier K Abulizi A Ainiwaer A Rehemuding R Ma X Ma YT. Unravelling the link between periodontitis and abdominal aortic calcification in the US adult population: a cross-sectional study based on the NHANES 2013–2014. BMJ Open. 2023;13:e068931. 10.1136/bmjopen-2022-068931 PMC10030668 36921940

[38] von Elm E Altman DG Egger M. The strengthening the reporting of observational studies in epidemiology (STROBE) statement: guidelines for reporting observational studies. Lancet. 2007;370:1453–7. 18064739 10.1016/S0140-6736(07)61602-X

[39] Yang S Zhao LS Cai C Shi Q Wen N Xu J. Association between periodontitis and peripheral artery disease: a systematic review and meta-analysis. BMC Cardiovasc Disord. 2018;18:141. 29980169 10.1186/s12872-018-0879-0 PMC6035462

[40] Ahn YB Shin MS Han DH. Periodontitis is associated with the risk of subclinical atherosclerosis and peripheral arterial disease in Korean adults. Atherosclerosis. 2016;251:311–8. 27450785 10.1016/j.atherosclerosis.2016.07.898

[41] Chen YW Umeda M Nagasawa T. Periodontitis may increase the risk of peripheral arterial disease. Eur J Vasc Endovasc Surg. 2008;35:153–8. 17964192 10.1016/j.ejvs.2007.08.016

[42] Çalapkorur MU Alkan BA Tasdemir Z Akcali Y Saatci E. Association of peripheral arterial disease with periodontal disease: analysis of inflammatory cytokines and an acute phase protein in gingival crevicular fluid and serum. J Periodontal Res. 2017;52:532–9. 27734498 10.1111/jre.12419

[43] Deng L Du C Liu L; “Flow and Toe” Research Team (FORT). Forecasting the global burden of peripheral artery disease from 2021 to 2050: a population-based study. Research (Washington, D.C.). 2025;8:0702. 40599301 10.34133/research.0702 PMC12209533

[44] Rizza S Cardellini M Martelli E. Occult impaired glucose regulation in patients with atherosclerosis is associated to the number of affected vascular districts and inflammation. Atherosclerosis. 2010;212:316–20. 20554281 10.1016/j.atherosclerosis.2010.05.017

[45] Lockhart PB Brennan MT Thornhill M. Poor oral hygiene as a risk factor for infective endocarditis-related bacteremia. J Am Dent Assoc. 2009;140:1238–44. 19797553 10.14219/jada.archive.2009.0046 PMC2770162

[46] Lockhart PB Brennan MT Sasser HC Fox PC Paster BJ Bahrani-Mougeot FK. Bacteremia associated with toothbrushing and dental extraction. Circulation. 2008;117:3118–25. 18541739 10.1161/CIRCULATIONAHA.107.758524 PMC2746717

[47] Bakhsh A Moyes D Mannocci F Proctor G Niazi S. Links between nosocomial endodontic infections and bacteremia associated with apical periodontitis and endodontic treatment. J Endod. 2025;51:140–9. 39581535 10.1016/j.joen.2024.11.009

